# Are the Best Worth the Wait? Physician Quality Scores and Access Delays

**DOI:** 10.1111/1475-6773.70148

**Published:** 2026-07-09

**Authors:** Yi‐Jung Shen, Samantha G. Auty, Kevin N. Griffith

**Affiliations:** ^1^ Department of Health Law, Policy and Management Boston University School of Public Health Boston Massachusetts USA; ^2^ Partnered Evidence‐Based Policy Resource Center, Boston Veterans Administration Healthcare System Boston Massachusetts USA; ^3^ Department of Health Policy Vanderbilt University Medical Center Nashville Tennessee USA; ^4^ VETeran's Wellbeing Through Innovation, Systems Science, and Experience in Learning Health Systems (VETWISE‐LHS), VA Tennessee Valley Healthcare System Nashville Tennessee USA

**Keywords:** access to care, pay‐for‐performance, quality of care, veterans, wait times

## Abstract

**Objective:**

The Veterans Health Administration's (VHA) Community Care Network generally includes physicians with higher Medicare Merit‐Based Incentive Payment System (MIPS) quality scores than nonparticipants, yet veterans disproportionately receive care from participating physicians with lower scores. Whether physician quality or availability influences veterans' use of community care has not been comprehensively evaluated. Our objective was to assess whether VHA community care specialists with higher MIPS quality scores receive greater referral volume or have longer wait times.

**Study Setting and Design:**

We used multivariate regression models to assess the associations between physicians' MIPS quality scores, ranging from 0 to 100, and wait times or referral volumes. We also tested whether wait time moderated the association between physician quality and referral volume.

**Data Sources and Analytic Sample:**

We used administrative data to identify referrals to community‐based specialists during 2021–2022. Referrals were then merged with MIPS quality scores from the Centers for Medicare & Medicaid Services using national provider identifiers.

**Principal Findings:**

Our sample included 83,911 specialty care referrals involving 43,736 specialists. The mean MIPS quality score was 81.9 (SD = 20.9), including 77.9% of physicians who scored ≥ 75, the threshold for a positive payment adjustment. The overall mean wait time was 21.8 days (SD = 22.0), and the mean referral volume was 6.6 per year (SD = 10.5). In covariate‐adjusted regression models, MIPS scores were not associated with wait times (+0.12 days per 10‐point increase, 95% CI: −0.02, 0.25) or referral volumes (−0.15 referrals per 10‐point increase, 95% CI: −0.37, 0.06). Wait time did not significantly moderate the relationship between MIPS score and referral volume.

**Conclusion:**

Physician quality appears to play a limited role in shaping referral decisions or access patterns in VHA community care. Although higher‐scoring physicians sometimes have slightly longer wait times, these differences are small and do not translate into higher referral volumes.

## Callout Box

- What is known on this topic.
  ○ The Veterans Health Administration's Community Care Network (CCN) includes many high‐quality specialists.
  ○ However, veterans disproportionately seek care from lower‐quality specialists within the CCN.
  ○ Public‐facing quality scores, such as Medicare's Merit‐Based Incentive Payment System (MIPS), may be important determinants of specialty care referral flows.
- What this study adds.
  ○ This is the first large‐scale analysis of the association between specialists' MIPS quality scores, mean new patient wait times, and mean annual referral volumes.
  ○ In both unadjusted and covariate‐adjusted analyses, we find that MIPS quality scores were generally not associated with wait times or referral volumes.
  ○ Wait times also did not moderate the association between MIPS quality scores or referral volumes.

## 1 Introduction

Healthcare performance metrics have proliferated with the goal of incentivizing quality improvement among providers and hospitals, enhancing patient autonomy through public‐facing scorecards, and supporting value‐based payment models [1, 2, 3]. One key initiative in this area is Medicare's Merit‐Based Incentive Payment System (MIPS), which scores healthcare providers on quality, cost, promoting interoperability, and improvement activities [4, 5, 6]. Under MIPS, clinicians may select from approximately 200 quality measures, including specialty‐specific measure sets [7]. For example, the otolaryngology specialty set includes 23 measures, such as appropriate antibiotic therapy for otitis media with effusion and adult sinusitis [6]. Clinicians are generally required to report at least six quality measures and can earn a positive adjustment through high performance, while lower scores or failure to report can result in a negative adjustment.

Research indicates that physicians may be motivated to improve care quality due to competitiveness, as they are influenced by perceptions of their performance relative to peers [8, 9]. However, studies also show that primary care physicians may not alter specialty care referral patterns based on quality information, tending instead to refer patients to specialists based on familiarity or specific patient needs [10]. In addition, there is ongoing debate about whether MIPS scores accurately reflect underlying care quality, as clinicians may selectively report measures from a broad menu, potentially introducing reporting and selection bias. Furthermore, potential score misattribution may occur if clinicians report through group practices or alternative payment models, where all affiliated providers receive identical scores regardless of individual performance. Notwithstanding these limitations, MIPS scores remain important to study because they are the most widely used and publicly available physician quality ratings in the United States and are accessible to consumers through the Center for Medicare & Medicaid Services' Care Compare tool. Lay consumers are unlikely to be aware of the academic literature documenting MIPS' methodological limitations, making it important to understand how these publicly available ratings influence referral patterns in practice.

The effects of quality ratings and scorecards on patient behavior have been primarily studied in hospitals and emergency rooms, with mixed results [8, 11, 12, 13, 14]. Some research suggests that patients prefer facilities with higher quality ratings, while other studies find minimal or no impact of hospital report cards on overall patient flows. The extent to which physician quality ratings influence patient choices remains unclear, especially regarding the potential for these ratings to cause congestion among higher‐quality physicians.

The U.S. Veterans Health Administration (VHA), serving over 9.1 million enrolled veterans and their families, provides a promising setting to explore these gaps. The Veterans Access, Choice, and Accountability Act of 2014, along with the VA MISSION Act of 2018, allow enrolled veterans to obtain healthcare from private providers under several circumstances, most commonly when travel distance or wait times for VHA services exceed specific limits or when community care is determined to be in the Veteran's best medical interest [15, 16, 17].

The VHA contracts with Optum Public Sector Solutions and TriWest Healthcare Alliance to utilize their extensive provider networks, including nearly half a million physicians, most of whom participate in Medicare [18]. VHA routinely evaluates these networks using standardized industry metrics, including Healthcare Effectiveness Data and Information Set (HEDIS) performance measures for clinical performance, the Survey of Healthcare Experience of Patients (SHEP) for patient experience, and contractor‐based assessments of network adequacy and provider performance [16, 17, 19]. These arrangements have facilitated the referral of millions of veterans to private sector specialists under the MISSION Act, internally referred to as “community care” [20].

Once referred to community care, veterans may choose an in‐network provider directly or request assistance from VHA referral coordination teams, who may book appointments on their behalf. While the VHA routinely monitors the quality of community care, quality is not assessed in the context of network adequacy standards [18], and it is not mandatory for referral coordinators to provide clinician quality ratings directly to veterans. Consequently, it remains unclear whether quality ratings significantly influence veterans' selection of community care providers. Notably, physicians participating in the VHA's community care network generally have higher CMS quality ratings than nonparticipants [21]. Yet, a recent study found that veterans disproportionately sought care from participating physicians with lower performance metrics [22].

To our knowledge, no comprehensive evaluation has been conducted on whether provider quality or availability influences veterans' use of community care providers. This study investigates whether community care specialists with higher quality scores experience higher referral volumes or longer wait times for new patients. We also examine whether new patient wait times moderate the relationship between referral volume and physician quality.

## 2 Methods

This study was considered exempt by the VA Boston Healthcare System Institutional Review Board and adheres to the Strengthening the Reporting of Observational Studies in Epidemiology (STROBE) reporting guideline for cross‐sectional studies. Institutional policy provides a waiver of informed consent because the research, which includes thousands of veterans, could not be practically carried out otherwise.

### 2.1 Data and Sample

We first obtained VHA administrative data on completed referrals to community‐based specialists during 2021–2022, including variables for wait times, specialty, and physician national provider identifier (NPI). We limited our sample to the 10 specialties with the highest referral volumes, including cardiology, dermatology, gastroenterology, neurology, otolaryngology, neurosurgery, ophthalmology, orthopedic surgery, podiatry, and urology. We also restricted the cohort to new patient referrals, excluding patients with a referral to the same specialty during the prior 3 years, consistent with VHA standards for defining new versus established patients [23].

Physician performance was measured using quality scores from CMS' Quality Payment Program data for 2021–2022 [24], which we linked to VHA data using NPIs. Patients may have multiple appointments and NPIs associated with a given VHA referral. Many of these NPIs were associated with diagnostic testing (e.g., radiologists, pathologists). Using data from the National Plan and Provider Enumeration System (NPPES), we selected the first individual (non‐group) NPI matching the requested specialty.

We excluded referrals to specialists with missing quality scores (12.6% of total). This missingness reflects MIPS eligibility and participation rather than data errors. Clinicians may lack scores if they opt not to participate, do not meet CMS low‐volume thresholds, are newly enrolled in Medicare, or participate in an advanced alternative payment model. The MIPS score is a weighted composite score that classifies physician quality as either positive (a bonus), neutral (no adjustment), or negative (a penalty) and adjusts their future Medicare reimbursement rates accordingly. Following previous work [22], we focused solely on the quality component, as the other domains may be less indicative of patient care [22]. A sample selection flow chart is provided in Figure S1.

### 2.2 Primary and Alternative Exposures

MIPS assigns participating clinicians an annual quality score that ranges from a minimum of 0 to a maximum of 100 points. The minimum score to avoid a penalty is 75 points [25]. In sensitivity analyses, we replaced the continuous MIPS quality score with a dichotomous indicator (≥ 75 vs. < 75) to assess potential differences between penalized and non‐penalized specialists.

### 2.3 Outcomes

Our primary outcomes included mean new patient wait times and total annual referral volumes. Wait times were defined as the timestamp difference between the date the appointment was scheduled and the appointment completion date for each community care referral [26]. Following previous work, appointments with wait times exceeding 365 days were removed to minimize data errors and improve overall data quality. We also winsorized records in the top 1% for referral volume, capping extreme values at the 99th percentile to reduce the influence of outliers [27].

### 2.4 Statistical Analysis

Our unit of analysis was the physician‐facility‐specialty‐year, based on the referring VA medical center. Our analysis proceeded in four steps. First, we calculated descriptive statistics for wait times and referral volumes stratified by MIPS quality score (< 75 vs. ≥ 75) and compared them using standardized mean differences (SMDs). We used SMDs rather than formal hypothesis tests to compare characteristics between MIPS score groups because statistical tests may identify trivial differences as significant in large samples. We interpreted absolute SMDs greater than 0.10 as meaningful group differences, consistent with commonly used thresholds in the balance diagnostics literature [28, 29].

Second, we produced binned scatter plots to illuminate trends in mean wait times and referral volumes across varying MIPS quality scores, both overall and for each specialty. Third, we estimated the associations between MIPS quality scores and each outcome using negative binomial regressions with year, specialty, and facility fixed effects. We report both an overall model and models stratified by specialty. Fourth, we assessed the potential moderating effect of wait times on the relationship between MIPS quality scores and referral volumes. To accomplish this, we reestimated the association between MIPS quality scores and referral volume, including an interaction term between each physician's score and mean wait time.

All analyses were conducted using Stata version 18. Each model incorporated analytic weights based on the number of referrals, with standard errors clustered by facility and specialist, and significance determined at the *α* = 0.05 level. To account for multiple comparisons, we applied Benjamini‐Hochberg corrections for all models and report both unadjusted confidence intervals and adjusted *p* values. Regression coefficients were converted to average marginal effets to aid in interpretbility.

## 3 Results

**FIGURE 1:**
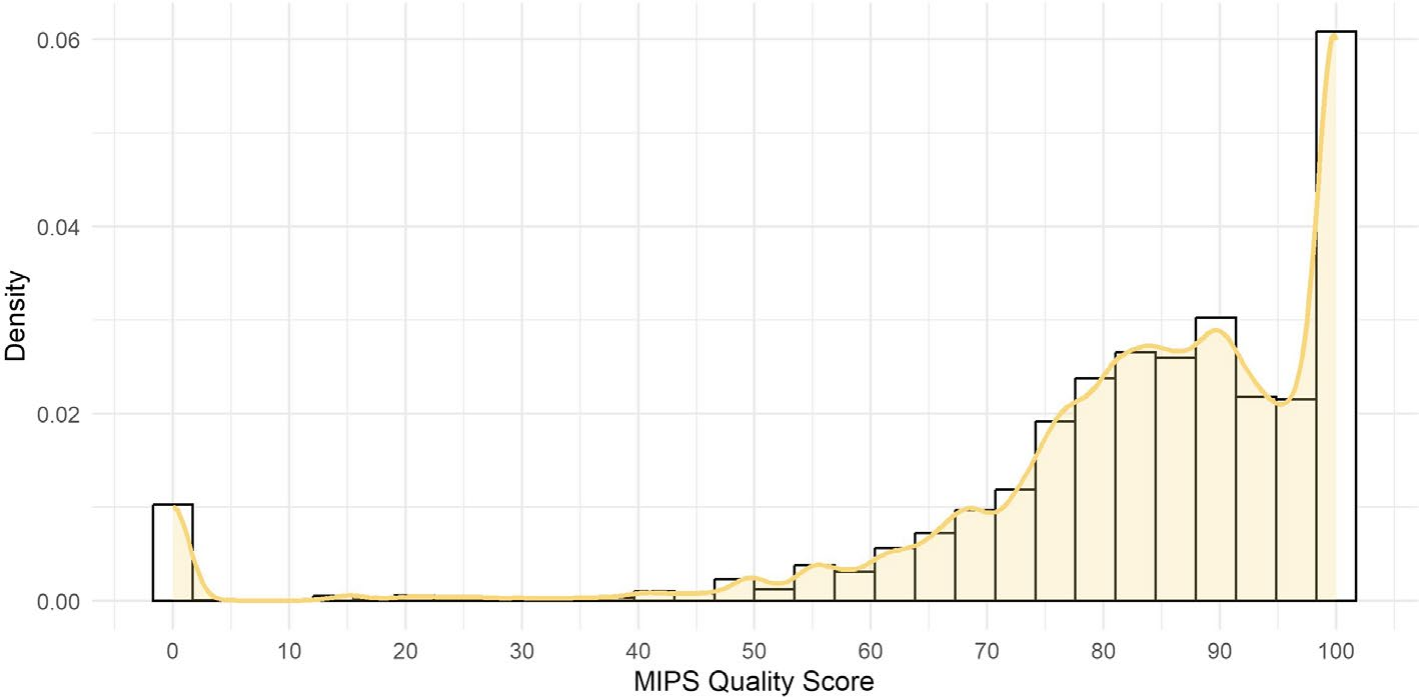
Density plot of MIPS Quality Scores. *Note:* The figure displays the density (proportion) of providers in 2.5‐point bins for quality scores and a smoothed line estimate for the overall distribution.

Our analytic sample included 83,911 specialty care referrals involving 43,736 specialists in 2021 and 2022. Overall, the mean MIPS quality score was 81.9 (SD = 20.9). A large majority of physicians received scores at or above 75 points (77.9%), while 17.8% received the maximum score of 100 points (Figure 1). Ophthalmologists in our sample had the highest mean MIPS quality score (88.2, SD = 19.2), while podiatrists had the lowest scores (69.6, SD = 33.7) (Table 1).

**TABLE 1:** Physician MIPS scores by specialty.

Specialty	*N*	MIPS score, mean (SD)	Wait time, mean (SD)				Referral volume, mean (SD)			
			Overall	MIPS score < 75	MIPS score ≥ 75	SMD	Overall	MIPS score < 75	MIPS score ≥ 75	SMD
Overall	83,911	81.9 (20.9)	21.8 (22.0)	21.7 (22.7)	21.9 (21.8)	0.01	6.6 (10.5)	6.9 (13.9)	6.9 (13.2)	0.00
Cardiology	15,436	82.5 (18.8)	20.1 (20.3)	20.4 (20.8)	20.0 (20.2)	0.02	5.1 (8.0)	5.1 (8.4)	5.1 (8.0)	0.01
Dermatology	7,166	84.1 (21.3)	27.0 (25.2)	28.6 (28.7)	26.5 (24.2)	0.08	8.1 (13.2)	7.1 (11.8)	8.4 (13.5)	0.10
Gastroenterology	7,017	82.1 (19.2)	26.6 (22.6)	25.8 (23.1)	26.8 (22.5)	0.05	6.3 (10.2)	6.6 (10.7)	6.2 (10.0)	0.04
Neurology	6,169	81.0 (19.9)	40.8 (36.0)	40.0 (36.6)	41.0 (35.8)	0.03	5.3 (9.3)	5.7 (10.1)	5.2 (9.1)	0.05
Otolaryngology	5,004	80.2 (21.2)	19.4 (17.6)	19.5 (18.2)	19.4 (17.4)	0.00	7.1 (11.3)	6.6 (10.8)	7.3 (11.4)	0.06
Neurosurgery	4,396	81.0 (17.7)	17.5 (15.3)	18.4 (15.9)	17.3 (15.1)	0.08	6.7 (10.6)	7.1 (10.8)	6.5 (10.5)	0.06
Ophthalmology	12,418	88.2 (19.2)	23.9 (21.1)	27.7 (25.2)	23.4 (20.4)	0.19	8.2 (12.0)	7.4 (11.6)	8.3 (12.0)	0.08
Orthopedic surgery	14,360	79.4 (20.6)	12.9 (12.9)	12.5 (11.8)	13.1 (13.2)	0.04	5.5 (8.6)	6.0 (9.5)	5.4 (8.3)	0.06
Podiatry	4,374	69.6 (33.7)	15.7 (14.2)	14.8 (13.2)	16.2 (14.8)	0.10	8.1 (12.6)	9.1 (13.9)	7.6 (11.7)	0.12
Urology	7,571	82.6 (17.5)	22.0 (18.4)	23.2 (20.0)	21.7 (17.9)	0.07	7.8 (11.4)	7.0 (10.8)	8.0 (11.6)	0.09

Abbreviations: MIPS, Merit‐Based Incentive Payment System; *N*, sample size; SD, standard deviation; SMD, standardized mean difference.

The overall mean wait time between referral initiation and appointment completion was 21.8 days (SD = 22.0). By specialty, the longest mean wait times were in neurology (40.8 days, SD = 36.0), while the shortest mean wait times were in orthopedic surgery (12.9 days, SD = 12.9). Observations referred to physicians with quality scores greater than or equal to 75 had slightly longer wait times compared to those with quality scores below 75 (21.9 vs. 21.7 days, SMD = 0.01), but these differences were not meaningful. However, wait times differed significantly by physician quality for ophthalmologists (MIPS quality score ≥ 75: 23.4 days; < 75: 27.7 days, SMD = 0.19) and podiatrists (MIPS quality score ≥ 75: 16.2 days; < 75: 14.8 days, SMD = 0.10).

Across our full sample, the mean number of referrals was 6.6 per year (SD = 10.5). Referral volumes for physicians with quality scores greater than or equal to 75 matched those with scores below 75 (6.9 vs. 6.9, SMD = 0.00). Ophthalmologists had the highest mean referral volume (8.2, SD = 12.0), while cardiologists had the lowest mean referral volume (5.1, SD = 8.0). Referral volumes differed significantly by physician quality for dermatologists (MIPS quality score ≥ 75: 8.4; < 75: 7.1, SMD = 0.10) and podiatrists (MIPS quality score ≥ 75: 7.6; < 75: 9.1, SMD = 0.12), but not for other specialties (Table 1).

**FIGURE 2:**
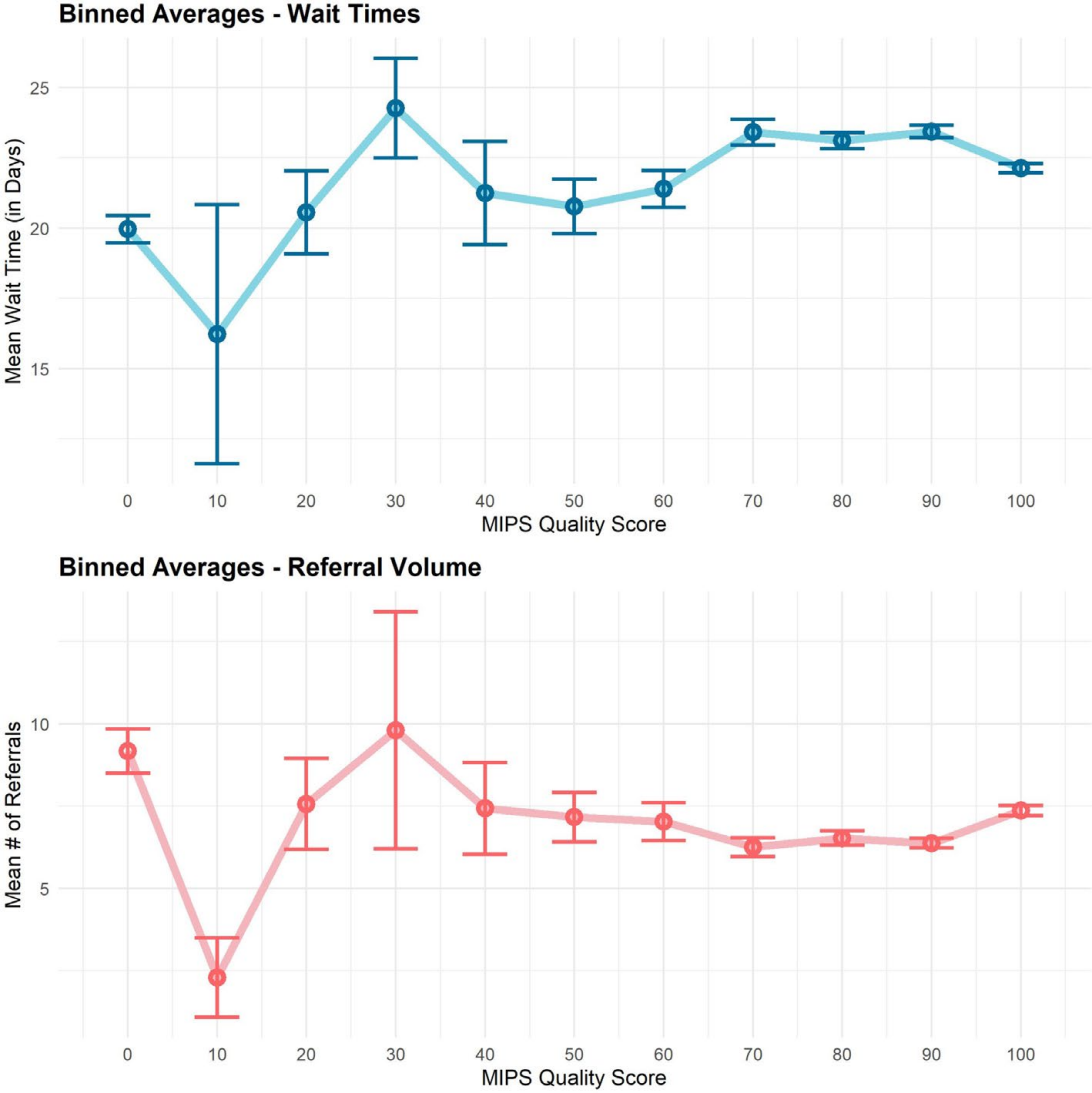
Binned scatter plots for the relationship between MIPS quality score, wait times, and referral volumes. *Note:* The figure displays overall binned scatter plots for the entire sample; specialty‐level data are provided in tabular format in the Appendix.

Overall, MIPS quality scores demonstrated a negligible positive correlation (*ρ* = 0.02) with wait times and a negligible negative correlation with referral volumes (*ρ* = −0.01) (Figure 2). MIPS quality scores were positively correlated with wait times in dermatology, gastroenterology, neurology, neurosurgery, and podiatry; negative correlations were observed among cardiology, ophthalmology, orthopedic surgery, and urology (Figure S2). For referral volume, positive correlations between MIPS quality scores were observed in dermatology, neurosurgery, and orthopedic surgery, and negatively correlated in the remaining specialties. Across the range of specialties and outcomes, Pearson correlation coefficients were small and ranged from −0.084 to 0.067 (Table S1).

In covariate‐adjusted regression models, a 10‐point increase in MIPS quality score was associated with an insignificant 0.12 day increase in wait times (95% CI: −0.02, 0.25) (Table 2). However, a 10‐point increase in MIPS quality score was associated with significantly increased wait times for dermatology (0.65 days; 95% CI: 0.23, 1.07) and podiatry (0.38 days; 95% CI: 0.16, 0.60). Results for cardiology, gastroenterology, neurology, otolaryngology, neurosurgery, ophthalmology, orthopedics, and urology were not statistically significant.

**TABLE 2:** Regression estimates for the association between MIPS quality score, wait times, and referral volumes.

	Wait time			Referral volume		
	Coef.	95% CI	Corrected *p*	Coef.	95% CI	Corrected *p*
Overall	0.12	(−0.02, 0.25)	0.308	−0.15	(−0.37, 0.06)	0.394
Cardiology	0.13	(−0.08, 0.34)	0.454	−0.15	(−0.68, 0.38)	0.646
Dermatology	0.65*	(0.23, 1.07)	0.022	0.22	(−0.56, 1.00)	0.646
Gastroenterology	0.14	(−0.40, 0.68)	0.646	−0.14	(−0.64, 0.37)	0.646
Neurology	0.82	(−0.17, 1.81)	0.308	−0.74	(−1.65, 0.17)	0.308
Otolaryngology	0.45	(0.08, 0.82)	0.127	0.25	(−0.36, 0.86)	0.623
Neurosurgery	0.25	(−0.19, 0.70)	0.495	0.89	(0.12, 1.66)	0.127
Ophthalmology	−0.25	(−0.61, 0.11)	0.394	−0.01	(−0.50, 0.48)	0.961
Orthopedic Surgery	0.06	(−0.16, 0.29)	0.646	−0.45	(−0.87, −0.02)	0.176
Podiatry	0.38*	(0.16, 0.60)	0.022	−0.20	(−0.64, 0.23)	0.564
Urology	−0.17	(−0.49, 0.15)	0.513	0.23	(−0.40, 0.86)	0.646

*Note:* Estimates were produced using negative binomial regression models with year, specialty, and facility fixed effects. Each model incorporated analytic weights based on the number of referrals, with standard errors clustered by facility and specialist. Regression coefficients were converted to average marginal effects; coefficients may be interpreted as the mean change in outcome per a 10‐point increase in MIPS score. *p* values were adjusted to account for the 22 multiple comparisons using the Benjamini–Hochberg method.

* *p* < 0.05.

** *p* < 0.01.

*** *p* < 0.001.

For referral volume, a 10‐point increase in MIPS quality score was not associated with referral volume overall or stratified by specialty. Furthermore, wait times did not significantly modify the association between MIPS quality score and referral volume in overall or stratified models (Table 3).

**TABLE 3:** Potential moderating effects of wait times on the association between MIPS quality scores and referral volumes.

Moderation effect (wait times)	Coef.	95% CI	Corrected *p*
Overall	−0.14	(−0.35, 0.07)	0.482
Cardiology	−0.15	(−0.66, 0.36)	0.688
Dermatology	0.37	(−0.35, 1.10)	0.576
Gastroenterology	−0.09	(−0.60, 0.41)	0.791
Neurology	−0.57	(−1.48, 0.34)	0.482
Otolaryngology	0.23	(−0.37, 0.84)	0.624
Neurosurgery	0.82	(0.06, 1.57)	0.242
Ophthalmology	0.03	(−0.44, 0.49)	0.908
Orthopedic Surgery	−0.44	(−0.87, 0.01)	0.242
Podiatry	−0.29	(−0.73, 0.14)	0.482
Urology	0.25	(−0.39, 0.90)	0.624

*Note:* Estimates were produced using negative binomial regression models with year, specialty, and facility fixed effects. Potential moderating effects were identified by the coefficient on the interaction between wait time and MIPS quality score. Each model incorporated analytic weights based on the number of referrals, with standard errors clustered by facility and specialist. Regression coefficients were converted to average marginal effects; coefficients may be interpreted as the mean change in outcome per a 10‐point increase in MIPS score. *p* values were adjusted to account for the 11 multiple comparisons using the Benjamini–Hochberg method.

* *p* < 0.05.

** *p* < 0.01.

*** *p* < 0.001.

Our regression results were largely consistent when MIPS quality scores were modeled as a binary variable (≥ 75 vs. < 75) instead of continuous (Table S2). We continued to observe a positive association between MIPS quality scores and wait times in podiatry (+2.18 days, 95% CI: 0.81, 3.55), while the estimate for dermatology increased in magnitude but lost significance. Consistent with our main findings, there were no significant associations between the MIPS payment adjustment threshold and referral volumes, either overall or in specialty‐stratified models.

## 4 Discussion

In this national cross‐sectional analysis, higher‐performing physicians were generally not associated with new patient wait times overall. However, veterans referred to higher‐performing dermatologists and podiatrists tended to experience longer wait times. Similarly, physician quality was not generally associated with referral volume for new patients. Furthermore, wait times did not meaningfully moderate the relationship between physician quality and referral volume across the highest‐volume specialties, reflecting little evidence of “congestion” effects around high‐quality providers.

The absence of either a consistent wait time‐quality or a volume‐quality relationship suggests that veterans are not responding to MIPS quality scores when choosing where to go for their care. While some veterans may prioritize factors such as driving distance, convenience, or specific medical needs, others may be unaware of the quality scores or where to locate them [15, 16]. In addition, under the Referral Coordination Initiative (RCI), VHA staff provide information and incorporate veterans' clinician preferences, which may shape where referrals are directed [30]. Our findings help to explain why, despite the presence of high‐quality providers in the VA's community care network (CCN) [21], veterans often seek care from those with lower MIPS scores [22].

The small differences and weak associations observed in this study may also reflect limitations of MIPS quality scores as both a measure of quality and a source of information for referral decision‐making. MIPS has several well‐documented limitations: it allows providers to voluntarily select a small subset of measures from a large menu, creating opportunity for selective reporting of measures on which they perform well; it may not capture equally meaningful dimensions of quality across all specialties examined; and scores may not be visible or salient to Veterans or VHA staff during the referral process. In addition, MIPS allows reporting at multiple organizational levels (individual clinician, group practice, and alternative payment model entities), which can result in score misattribution. However, our research question examines whether the scores as displayed to veterans and referral coordinators through Care Compare influence referral decisions, regardless of how those scores were constructed.

Collectively, these limitations suggest that MIPS scores may be more reflective of a provider's willingness and ability to report quality data than their actual clinical performance. Therefore, referral decisions may be shaped more by provider availability, geographic proximity, prior patient or clinician preferences, and information provided through referral coordination than by MIPS performance. These patterns also highlight an opportunity to enhance how clinician quality information is integrated into referral workflows and presented to both coordinators and veterans. Strengthening the role of quality metrics in referral decision‐making could enhance veteran autonomy, help ensure that veterans have access to timely, high‐quality specialty care, and further advance the VA's mission to deliver value‐driven, veteran‐centered care.

## 5 Limitations

Our study has several limitations. First, participation in MIPS is mandatory for most clinicians but is voluntary for those with low billing volumes or who participate in an advanced alternative payment model. Thus, our results may not generalize to nonparticipants. Second, the quality measures that participating providers report on are self‐selected, which may not reflect important dimensions of care quality or patient experience [5, 31]. Third, we could not observe providers who participate in the VHA's CCN but received no referrals, so they were not included in these analyses.

## 6 Conclusion

Physician quality appears to play a limited role in shaping referral decisions or access patterns in VA community care. Although higher‐quality physicians often have longer wait times, these differences are small in magnitude and do not translate into systematically higher referral volumes. Efforts to integrate quality metrics more explicitly into community care referral workflows, coupled with transparency for veterans and coordinators, could help ensure that access expansion through the MISSION Act is matched by consistent quality of care.

## Funding

This study was funded by Veterans Affairs Quality Enhancement Research Initiative (grant PEC 16‐001).

## Conflicts of Interest

The authors declare no conflicts of interest.

## Supporting information


**Table S1:** Pearson correlations between MIPS Quality Scores, wait times, and referral volumes.
**Table S2:** Regression estimates for the association between a binary measure of MIPS quality score (≥ 75 vs. < 75), wait times, and referral volumes.
**Figure S1:** Sample selection flow chart.
**Figure S2:** Binned scatter plots for the relationship between MIPS quality score, wait times, and referral volumes, by individual specialty.

## Data Availability

Research data are not shared.
