# Supplementary material for: Are the Best Worth the Wait? Physician Quality Scores and Access Delays
Source: Health Serv Res. 2026 Jul 9;61(4):e70148. doi: 10.1111/1475-6773.70148 (PMC13347620; doi:10.1111/1475-6773.70148)
Supplement: Supplementary file 1 — Table S1: Pearson correlations between MIPS Quality Scores, wait times, and referral volumes. Table S2: Regression estimates for the association between a binary measure of MIPS quality score (≥ 75 vs. < 75), wait times, and referral volumes. Figure S1: Sample selection flow chart. Figure S2: Binned scatter plots for the relationship between MIPS quality score, wait times, and referral volumes, by individual specialty. [file HESR-61-e70148-s001.docx]

**Supplementary Online Content**

**Supplementary Table 1. Pearson correlations between MIPS Quality Scores, wait times, and referral volumes**

**Supplementary Table 2. Regression Association of Physician MIPS Quality Scores and Wait Time and Referral Volume**

**Supplementary Figure 1.** **Sample selection flow chart**

**Supplementary Figure 2. Binned scatter plots for the relationship between MIPS quality score, wait times, and referral volumes, by individual specialty**

**Supplementary Table 1. Pearson correlations between MIPS Quality Scores, wait times, and referral volumes**

|  | **Wait time** | **Referral volume** |
| --- | --- | --- |
|  | **Coef.** | **Coef.** |
| **Overall** | 0.024 | -0.010 |
| **Cardiology** | -0.023 | -0.006 |
| **Dermatology** | 0.009 | -0.001 |
| **Gastroenterology** | 0.003 | -0.012 |
| **Neurology** | 0.067 | -0.044 |
| **Otolaryngology** | 0.017 | 0.008 |
| **Neurosurgery** | -0.009 | -0.010 |
| **Ophthalmology** | -0.048 | 0.013 |
| **Orthopedic Surgery** | -0.015 | -0.031 |
| **Podiatry** | 0.035 | -0.069 |
| **Urology** | -0.084 | 0.000 |

**Supplementary Table 2. Regression estimates for the association between a binary measure of MIPS quality score (**≥**75 vs <75), wait times, and referral volumes**

|  | **Wait time** | | |  | **Referral volume** | | |
| --- | --- | --- | --- | --- | --- | --- | --- |
|  | **Marginal Effect** | **95% CI** | **Corrected P-value** |  | **Marginal Effect** | **95% CI** | **Corrected P-value** |
| **Overall** | 0.19 | (-0.43, 0.81) | 0.699 |  | -0.17 | (-1.37, 1.04) | 0.826 |
| **Cardiology** | 0.30 | (-0.58, 1.19) | 0.696 |  | 0.42 | (-1.90, 2.73) | 0.798 |
| **Dermatology** | 1.25 | (-1.28, 3.79) | 0.609 |  | 5.62 | (1.74, 9.51) | 0.055 |
| **Gastroenterology** | 1.58 | (-0.41, 3.57) | 0.330 |  | -1.33 | (-4.65, 1.99) | 0.696 |
| **Neurology** | 0.85 | (-3.03, 4.73) | 0.771 |  | -1.37 | (-5.34, 2.60) | 0.696 |
| **Otolaryngology** | 1.42 | (-0.44, 3.28) | 0.330 |  | 2.34 | (-0.69, 5.36) | 0.330 |
| **Neurosurgery** | 0.01 | (-1.91, 1.93) | 0.993 |  | 3.37 | (-0.26, 7.01) | 0.330 |
| **Ophthalmology** | -1.94 | (-4.04, 0.17) | 0.330 |  | -0.78 | (-4.15, 2.58) | 0.771 |
| **Orthopedic Surgery** | 0.34 | (-0.67, 1.36) | 0.696 |  | -1.61 | (-3.41, 0.19) | 0.330 |
| **Podiatry** | **2.18*** | **(0.81, 3.55)** | **0.044** |  | -2.95 | (-6.50, 0.59) | 0.330 |
| **Urology** | -0.83 | (-2.23, 0.57) | 0.486 |  | 1.65 | (-1.00, 4.30) | 0.486 |

**Notes:** Estimates were produced using ordinary least squares regression models with year, specialty, and facility fixed effects. Our primary predictor was a binary indicator taking on a value of one if MIPS quality scores met or exceed 75 points, zero otherwise. Each model incorporated analytic weights based on the number of referrals, with standard errors clustered by facility and specialist. P-values were adjusted to account for the 22 multiple comparisons using the Benjamini-Hochberg method; *p<.05 **p<.01 ***p<.001.

**Supplementary Figure 1. Sample selection flow chart**

All community care consult from 2021-2022

5,333,528 referrals

New patient consults for top ten specialists

1,656,808 referrals

Excluding patients with a referral to the same specialty during the prior three years

New patient consults

4,645,005 referrals

Remaining ten specialties with the highest referral volumes

Referrals had claimed in community care

1,469,080 referrals

Selecting the first physician matching the requested specialty

837,109 referrals

Removing MIPS quality scores missing

Physicians with complete MIPS quality scores

648,578 referrals

Final sample 539,055 referrals

Aggregated to physician-specialty-facility-year level

83,911 observations

Removing anomalous wait time cases

**Supplementary Figure 2. Binned scatter plots for the relationship between MIPS quality score, wait times, and referral volumes, by individual specialty**

**(a) Cardiology**

**
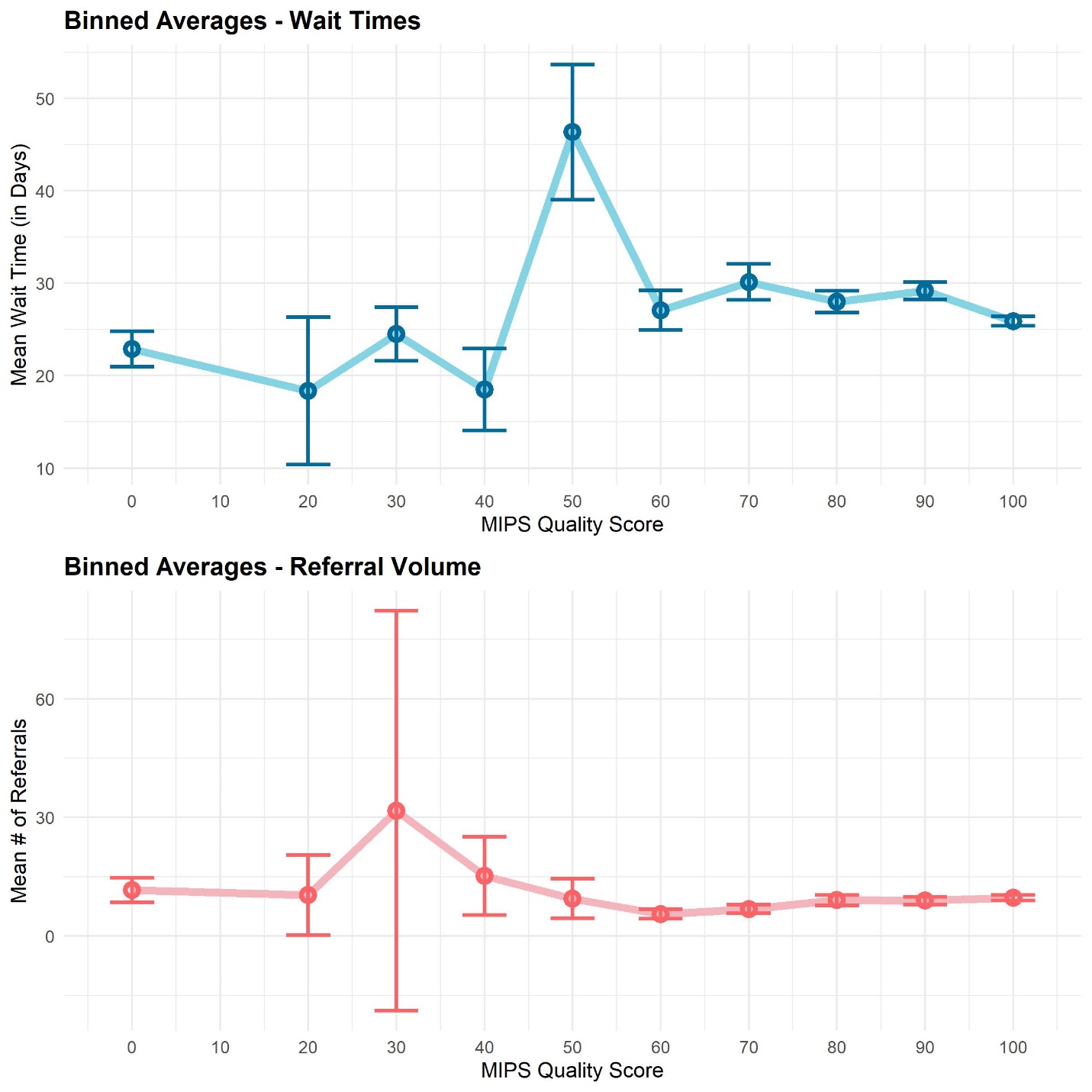
**

**(b) Dermatology**

**
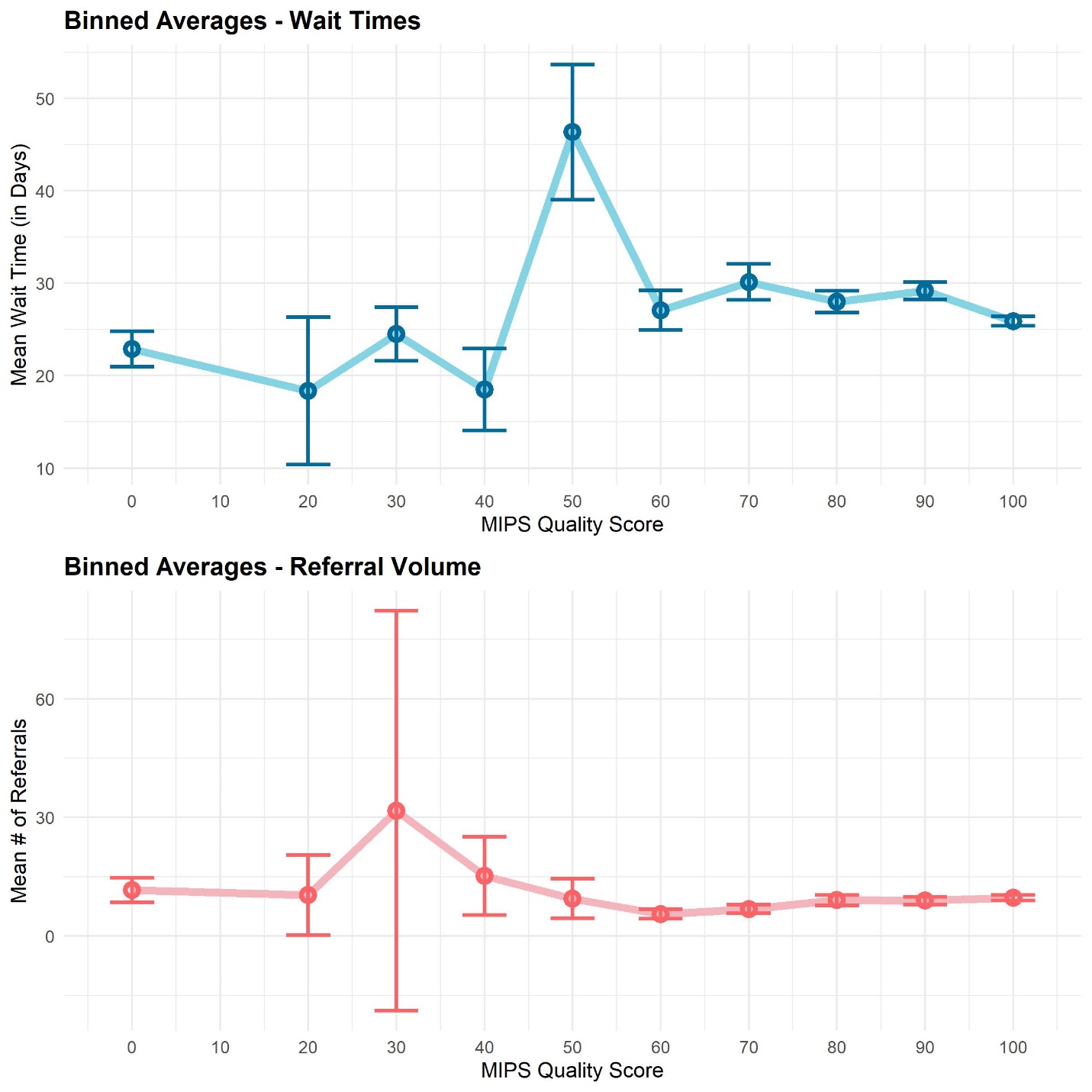
**

**(c) Gastroenterology**

**
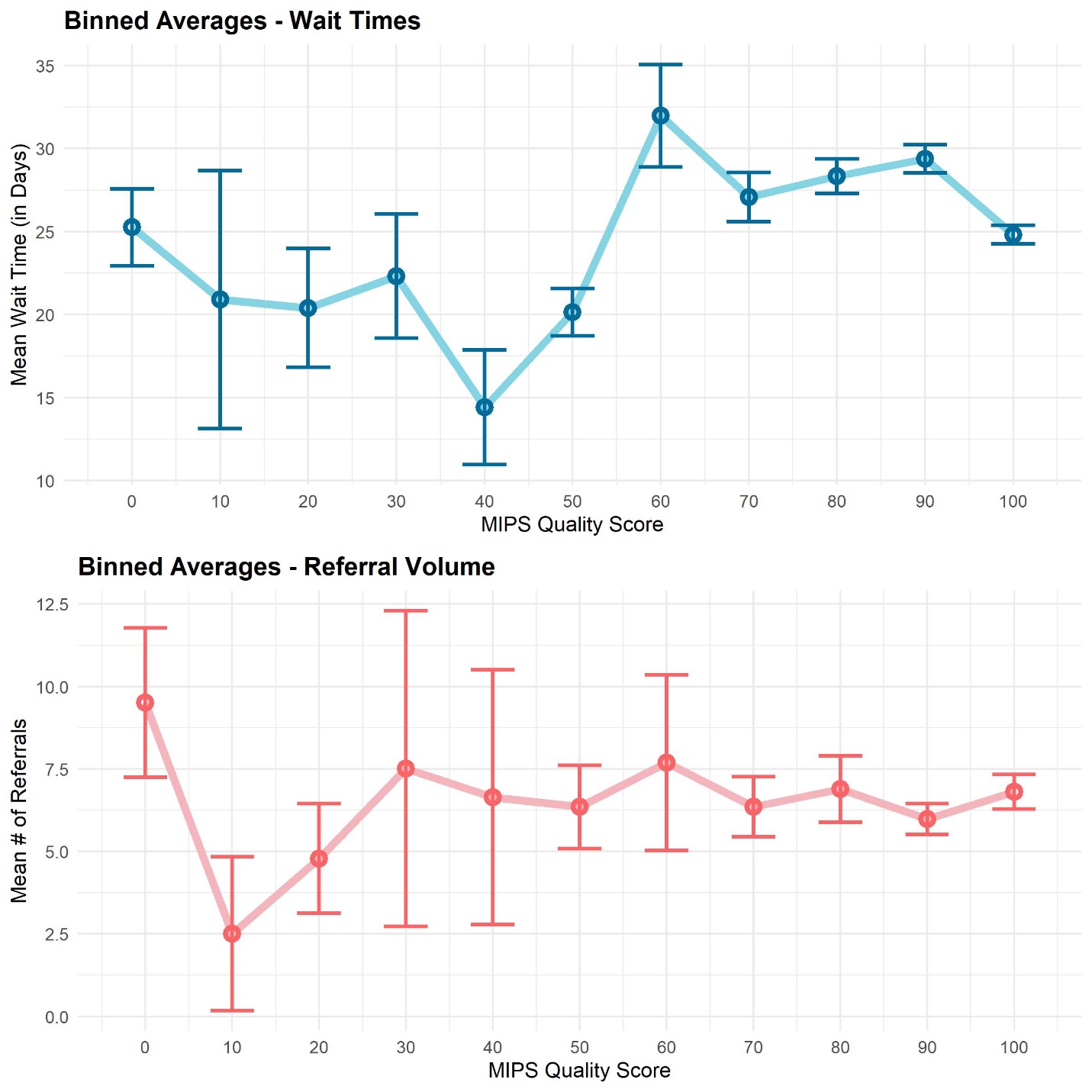
**

**(d) Neurology**

**
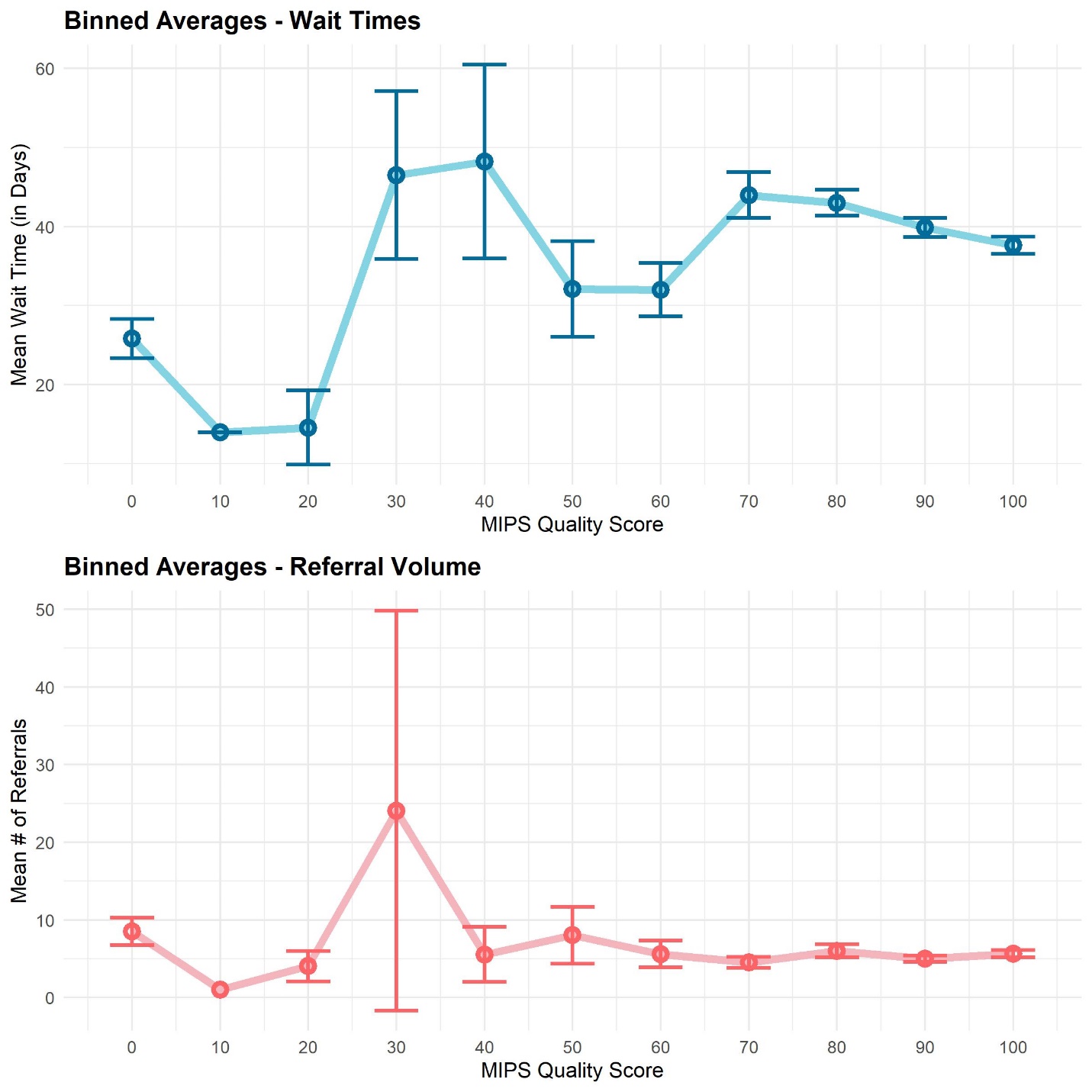
**

**(e) Neurosurgery**

**
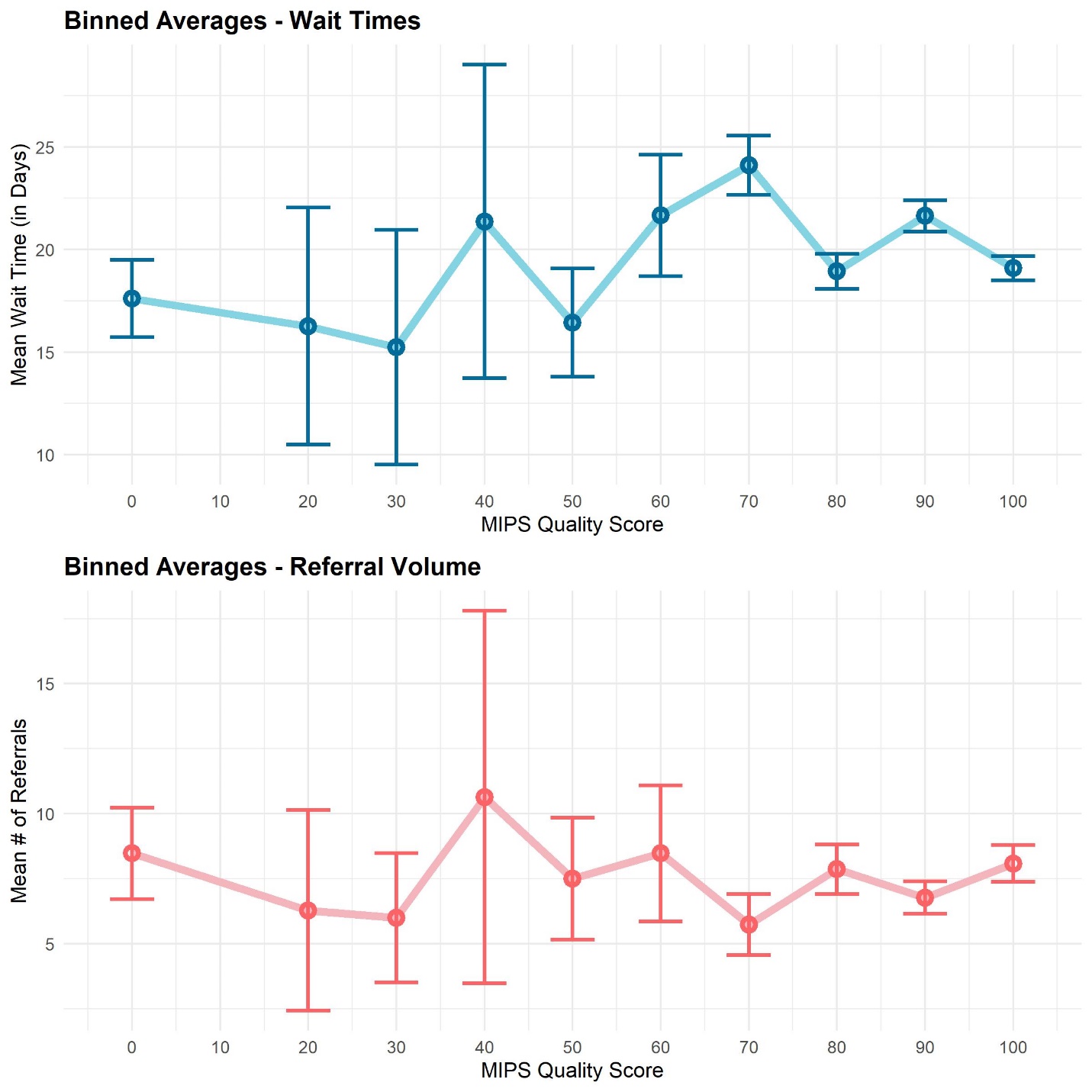
**

**(f) Ophthalmology**

**
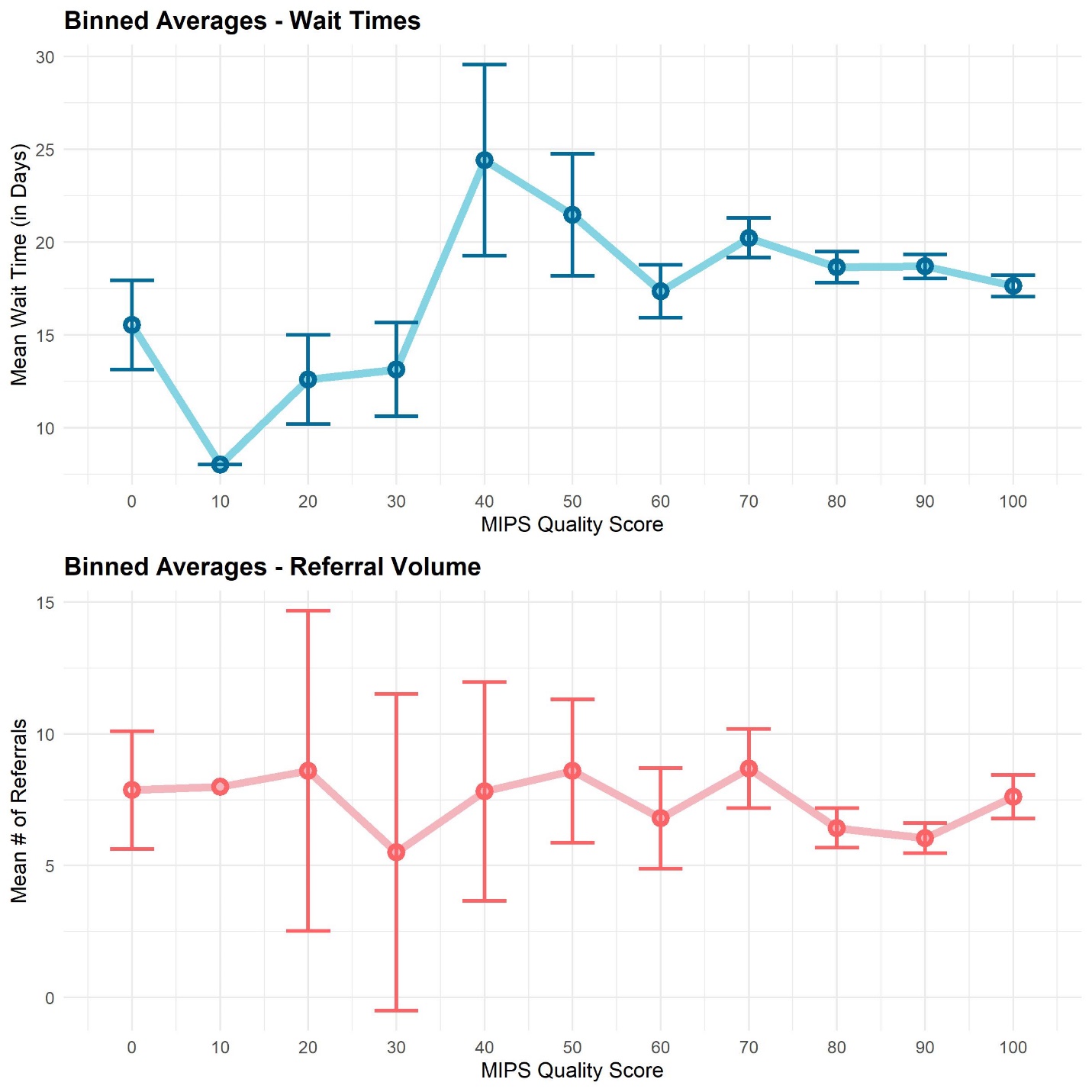
**

**(g) Orthopedic Surgery**

**
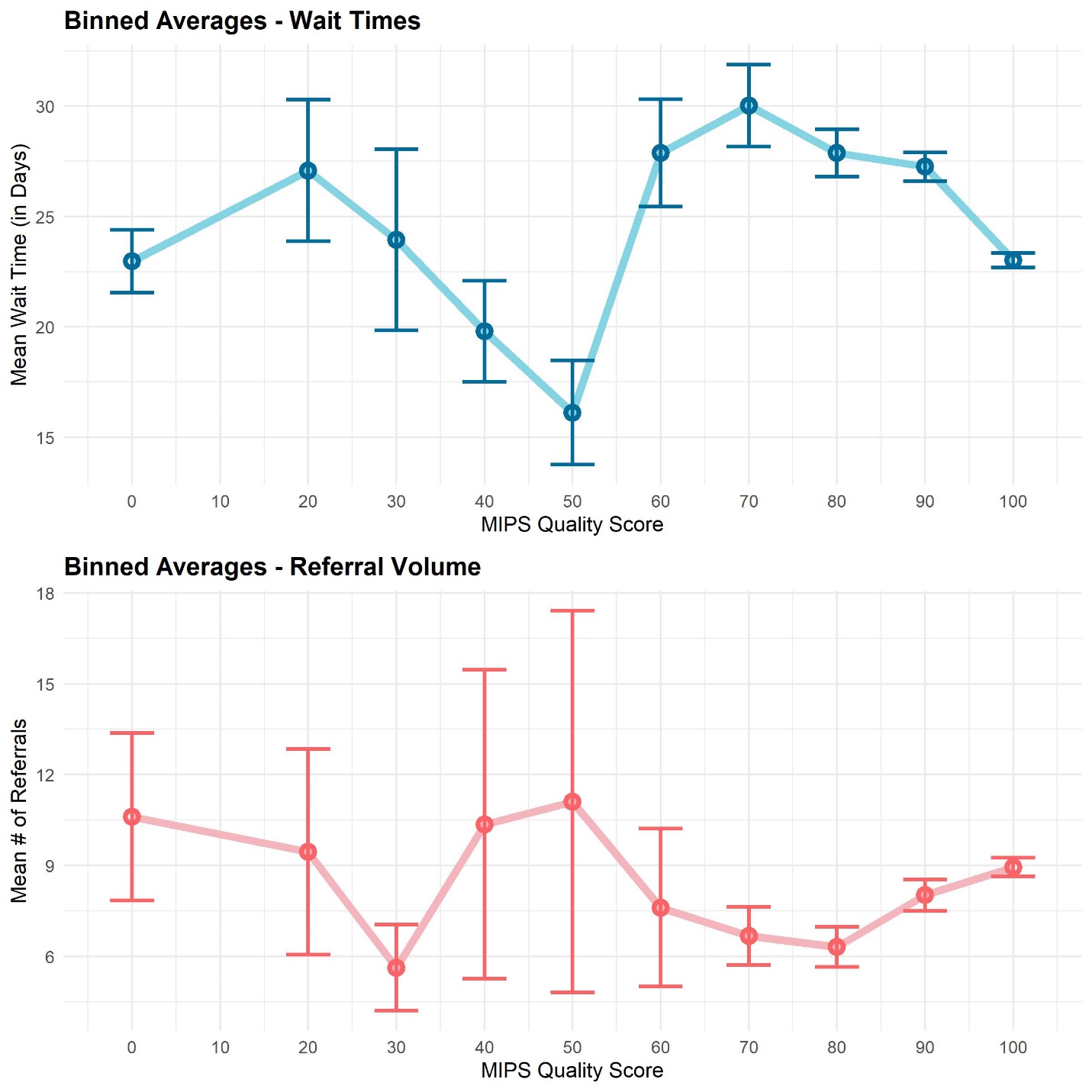
**

**(h) Otolaryngology**

**
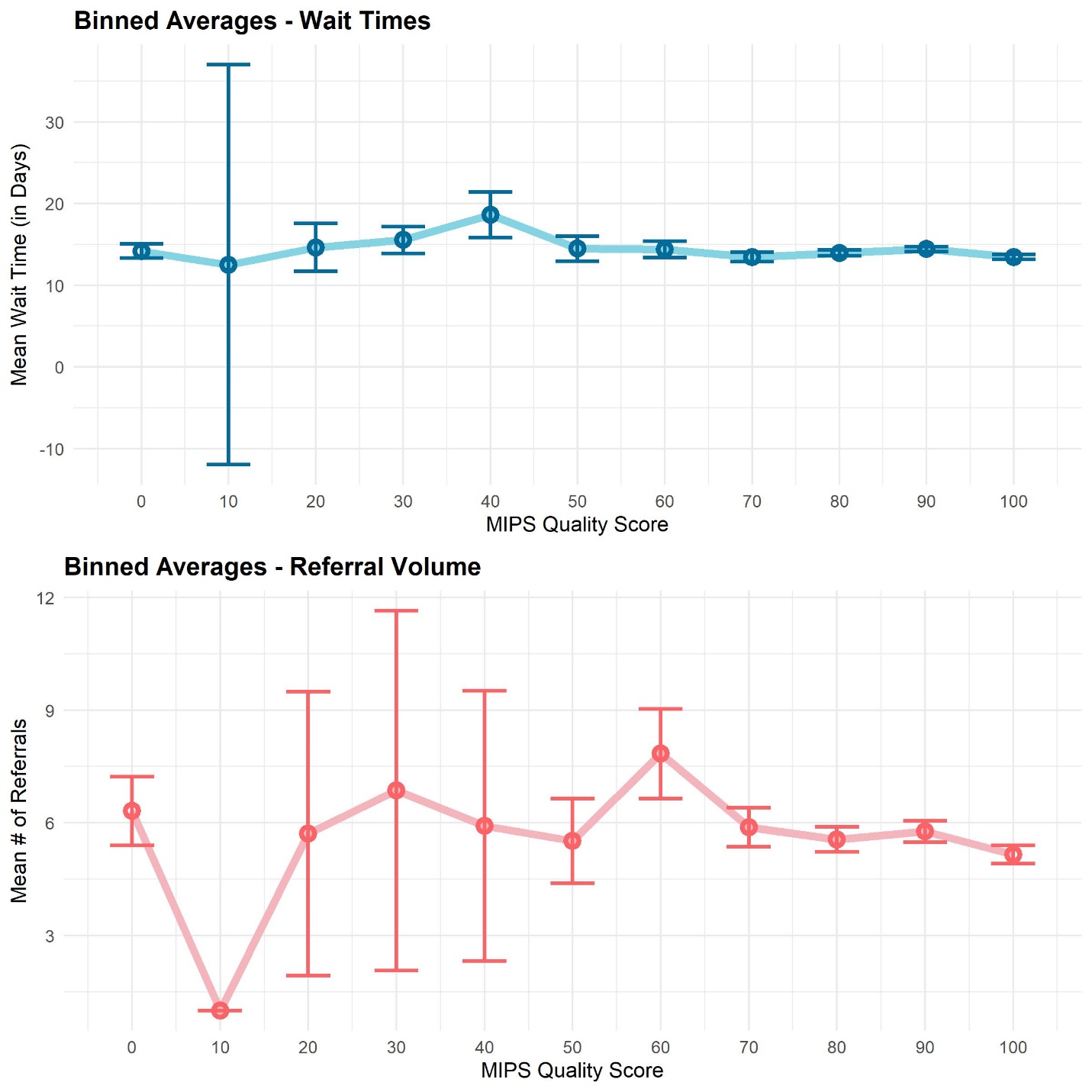
**

**(i) Podiatry**

**
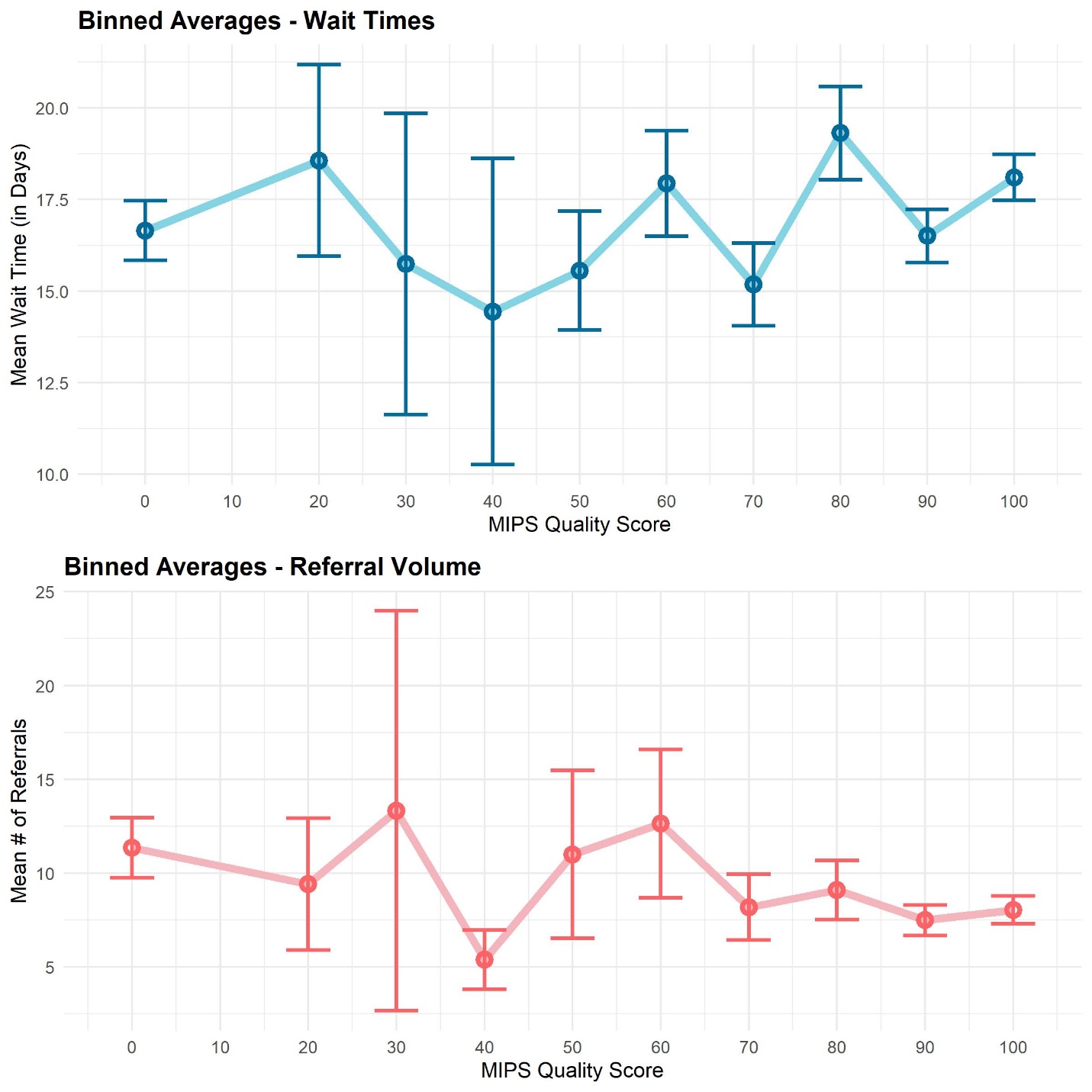
**

**(j) Urology**

**
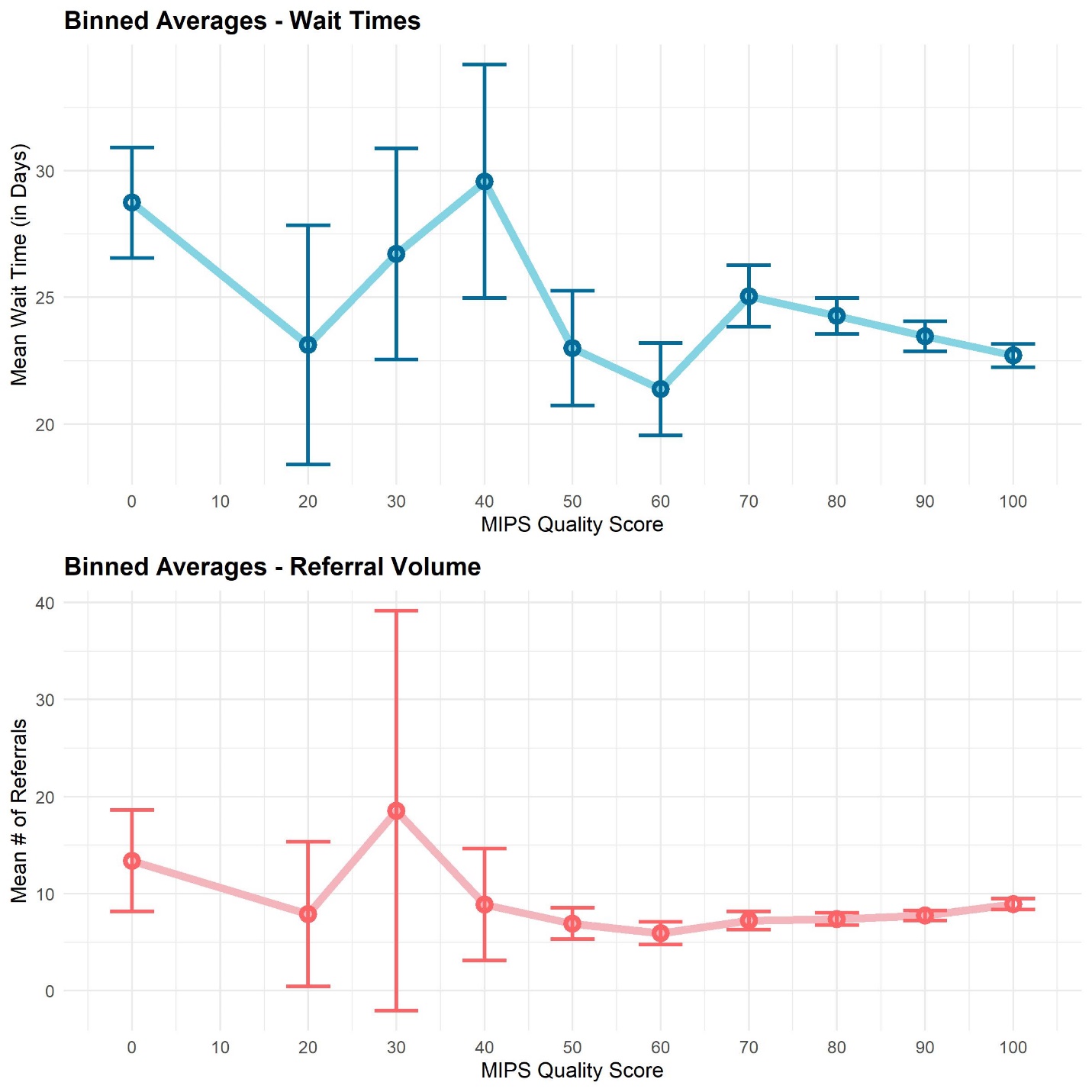
**
